# Sensor Level Functional Connectivity Topography Comparison Between Different References Based EEG and MEG

**DOI:** 10.3389/fnbeh.2018.00096

**Published:** 2018-05-15

**Authors:** Yunzhi Huang, Junpeng Zhang, Yuan Cui, Gang Yang, Qi Liu, Guangfu Yin

**Affiliations:** ^1^College of Electrical Engineering and Information Technology, Sichuan University, Chengdu, China; ^2^College of Materials Science and Engineering, Sichuan University, Chengdu, China; ^3^Computer Teaching and Research Section, Chengdu Medical College, Chengdu, China

**Keywords:** functional connectivity topography, MEG, EEG, reference schemes of EEG, sensor level, face-recognition

## Abstract

Sensor-level functional connectivity topography (sFCT) contributes significantly to our understanding of brain networks. sFCT can be constructed using either electroencephalography (EEG) or magnetoencephalography (MEG). Here, we compared sFCT within the EEG modality and between EEG and MEG modalities. We first used simulations to look at how different EEG references—including the Reference Electrode Standardization Technique (REST), average reference (AR), linked mastoids (LM), and left mastoid references (LR)—affect EEG-based sFCT. The results showed that REST decreased the reference effects on scalp EEG recordings, making REST-based sFCT closer to the ground truth (sFCT based on ideal recordings). For the inter-modality simulation comparisons, we compared each type of EEG-sFCT with MEG-sFCT using three metrics to quantize the differences: Relative Error (RE), Overlap Rate (OR), and Hamming Distance (HD). When two sFCTs are similar, RE and HD are low, while OR is high. Results showed that among all reference schemes, EEG-and MEG-sFCT were most similar when the EEG was REST-based and the EEG and MEG were recorded simultaneously. Next, we analyzed simultaneously recorded MEG and EEG data from publicly available face-recognition experiments using a similar procedure as in the simulations. The results showed (1) if MEG-sFCT is the standard, REST—and LM-based sFCT provided results closer to this standard in the terms of HD; (2) REST-based sFCT and MEG-sFCT had the highest similarity in terms of RE; (3) REST-based sFCT had the most overlapping edges with MEG-sFCT in terms of OR. This study thus provides new insights into the effect of different reference schemes on sFCT and the similarity between MEG and EEG in terms of sFCT.

## Introduction

Functional connectivity (FC) is utilized to measure functional coupling and interaction between different brain regions. Usually, topographical FC patterns can be conducted on sensor level or on source level. Unlike source level FC analysis, sensor level FC analysis directly takes use of scalp surface measured EEG/MEG recordings and is still widely used. Sensor-level functional connectivity topography (sFCT) is regarded as a non-invasive and effective approach for approximating the relationship among cortex areas (De Vico Fallani et al., 2014; Wang et al., 2014; Garces et al., 2016) and it has been playing an important role in assisting physicians to diagnose related diseases (Schoonheim et al., 2013; Toussaint et al., 2014; Min et al., 2015; Sato et al., 2017). Generally, surficial electroencephalogram (EEG) or magnetoencephalotopography (MEG) can be used to construct and analyze sFCT (Stam, 2004; Schindler et al., 2008). The two measurements have their own unique advantages and disadvantages, and getting full use out of them is a challenging issue. Based on the intrinsic relationship between electrical field and magnetic field, simultaneously collected MEG and EEG should have a certain degree of similarity in term of sFCT because both originate from neuronal activity within brain. sFCT might therefore be an effective means to explore the relationship between MEG-derived sensor-level neural networks and those constructed by differing EEG reference schemes.

Before investigating the relationship between MEG—and EEG-sFCT (inter-modality) gaining an insight into the effect of EEG reference on sFCT (intra-modality) is imperative. EEG measures differences in scalp potential, which are derived from ohmic currents induced by electrical brain activity. The scalp potential is a relative value and the potential of a point always depends on where the reference point is set. Choosing an appropriate EEG reference has a significant influence on brain-activity measurements and allows more accurate cross-study comparisons (Rummel et al., 2007; Kayser and Tenke, 2010). During the past several decades, numerous studies have developed different EEG reference schemes and attempted to determine which one is the most suitable (Fein et al., 1988; Dien, 1998; Mima and Hallett, 1999; Zaveri et al., 2000, 2006; Hagemann et al., 2001; Rummel et al., 2007). Because a constant, zero reference does not exist in the human body (Geselowitz, 1998; Nunez and Srinivasan, 2006), non-zero activity is always present regardless of which reference scheme is chosen. A “good” EEG reference should be the most similar with the ideal measurement. Preferences for EEG reference schemes vary across laboratories, often depending on research fields, and clinical practices (Kayser and Tenke, 2010; Nunez, 2010). In this study, we chose the following four widely used EEG reference schemes to construct EEG-based sFCT: (1) the reference electrode standardization technique (REST) (Yao, 2001; Zhai and Yao, 2004; Yao et al., 2005, 2007) (2) the average reference (AR, i.e., average potential over all EEG electrodes) (Offner, 1950; Nunez et al., 2001), (3) linked mastoids (LM) (Gevins and Smith, 2000; Croft et al., 2002), and (4) the left-ear reference(LR) (Basar et al., 1998; Thatcher et al., 2001). The infinity reference (IR), which is considered to be located rather far away and to deliver the least interruptions to the recording electrodes, is usually regarded as a zero-reference that provides a static ground truth (Yao, 2001; Zhai and Yao, 2004) for evaluating different EEG references.

Unlike EEG, MEG depends on the magnetic field outside the head, which is induced by current flow within the brain (Darvas et al., 2004; Baillet, 2017). MEG has several prominent advantages over EEG. It is reference free, and the effect of volume conduction is negligible (van den Broek et al., 1998). However, MEG is much more expensive than EEG and the cost prohibits widespread utilization.

Under different pathological or physiological conditions, a better understanding of disease can be obtained through comparing the similarity of the two modalities (MEG and EEG) on sFCT. The overall structure of this study is as follows; first, with simulated data, we explore the similarity of intra-modality (within EEG references) and inter-modality (EEG and MEG) by calculating sFCT based on imaginary part of coherency (IC) (Nolte et al., 2004). Particularly, three metrics are exploited to evaluate the differences among different sFCTs; (1) Relative Error (RE), (2) Overlap Rate (OR) (3) Hamming Distance (HD) (Makram Talih, 2005; Medkour et al., 2010; van Wijk et al., 2010). Following this, real data is applied to further validate the performance of each EEG reference scheme and the connection between MEG and EEG on sFCT.

## Materials and methods

### sFCT construction

#### Imaginary part of coherency

Volume conduction is a phenomenon that affects the coherency between EEG channels, making artifacts difficult to distinguish from true brain activity (Chella et al., 2016). The imaginary part of coherency (IC) has proved to be helpful in extracting the true functional connectivity between EEG electrodes and making it less effected by volume conduction (Nolte et al., 2004; Schoffelen and Gross, 2009). Here, we briefly recalled the definition of coherency.

Assuming *v*_*i*_(*f*) and *v*_*j*_(*f*) represent the complex-form Fourier transforms of time series signals ${\hat{v}}_{i}\left(t\right)$ and ${\hat{v}}_{j}\left(t\right)$, respectively, and the two signals are recorded from the EEG electrodes which are indexed by *i* and *j*, with any given reference. Their cross-spectrum is defined as follows.

(1) $$\begin{array}{l} S_{ij}\left(f\right)=\langle v_{i}\left(f\right)v_{j}^{*}\left(f\right)\rangle \end{array}$$

where, ^*^denotes the complex conjugation, and 〈〉 denotes expectation value. Coherency is defined as the cross-spectrum normalized by power,

(2) $$\begin{array}{l} Coh_{ij}\left(f\right)=\frac{S_{ij}\left(f\right)}{{\left(S_{ii}\left(f\right)S_{jj}\left(f\right)\right)}^{1/2}} \end{array}$$

The imaginary part of coherency among all the electrodes/sensors of EEG/MEG is used to construct sFCT.

#### Construction of sFCT

Based on the IC matrix, sFCTs are constructed to describe the connectivity among multiple regions or nodes, and to reflect the synchronization and the interaction among brain regions. To enable a better representation of the topography of network connectivity, weak links between nodes are removed using a binary network that sets a connectivity threshold. The binary network is determined by the following steps: first, according the weights of edges, all the edges were sorted in descending order; second, the largest x (x refers to network sparsity, ranging from 20 to 80, and the step width was set to 10) weights in the IC matrix were selected, accordingly, the minimum value of the selected largest x weights was taken as the threshold to construct a binary network; finally, the nodes with larger values than the threshold were set to 1, and the rest nodes were set to 0. In this way, the binary network was generated. Here, a series of threshold values were placed on the sFCTs, which yielded different network sparsities. This allowed us to observe and validate the robustness of different sFCTs.

### MEG based sFCT construction

**Figure 1:**
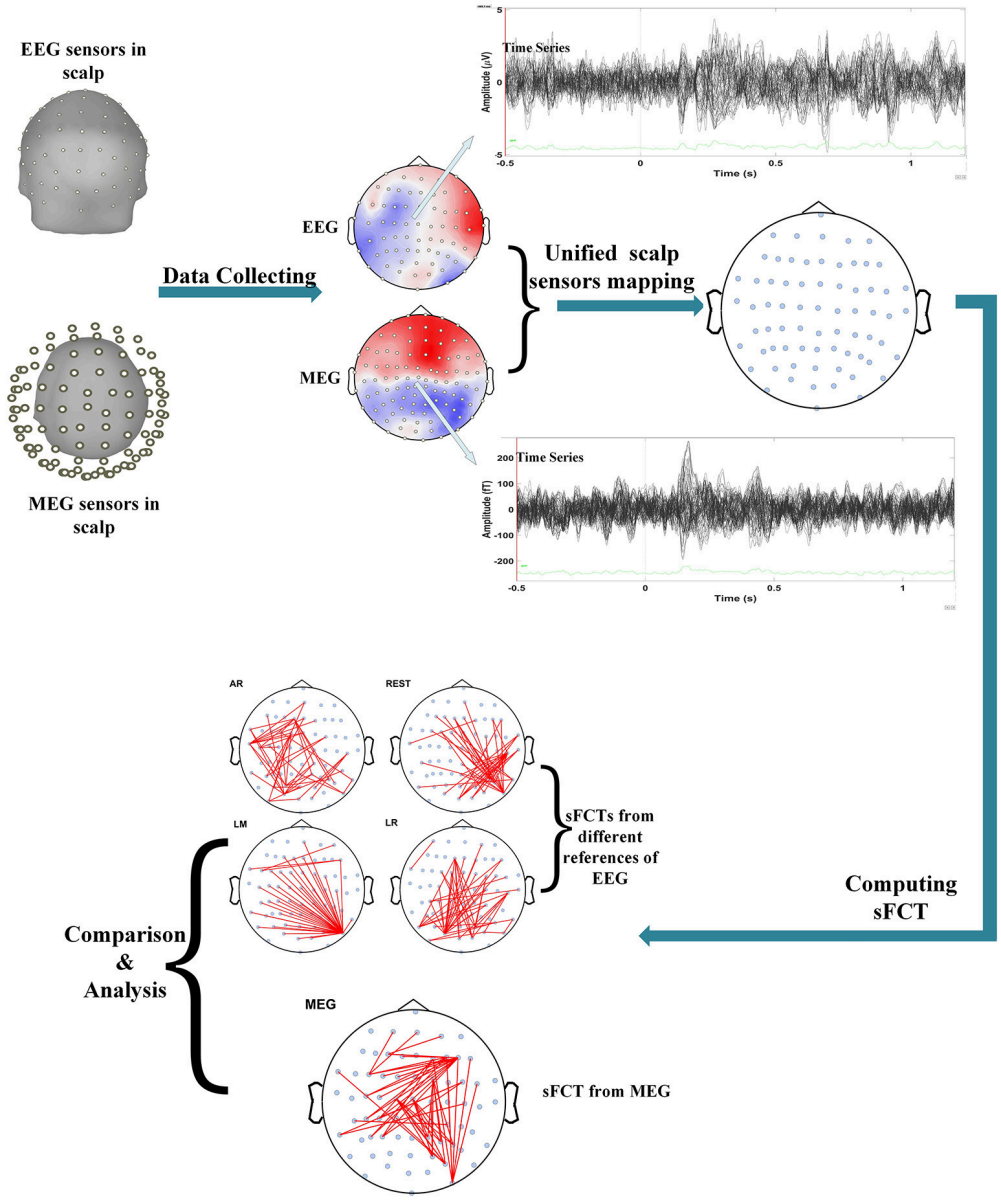
Flowchart depicting the procedure for real data. Processing included several important steps. First, data is set so that scalp MEG collection and scalp EEG collection come from the same stimulus. Second, electrode transformations are conducted by unifying the EEG and MEG sensors on the scalp. Then, sFCTs are computed for each EEG reference scheme and for MEG. Finally, the differences between methods are analyzed with quantized metrics.

We performed several operations when processing and analyzing the real MEG data, such as unifying scalp-sensor mapping, computing sFCTs, and analyzing and comparing sFCTs based on the different methods (seen in Figure 1). Inter-modality comparisons of sFCTs can only be computed when the modalities share the same electrode distribution. Therefore, the unifying procedure is essential for subsequent procedures. In this study, we transformed the MEG signal so that it seems that the MEG and EEG data are collected from the same location by the following steps.

(a)With the least square method, the coordinates of the MEG sensors and EEG electrodes were exploited to fit the spherical radius of MEG and EEG, respectively. The fit radii were written as *r*_*eeg*_ and *r*_*meg*_, respectively.

(b)Based on the spherical radii of the MEG sensors and EEG electrodes from step (a), the MEG sensors were mapped to the sphere fitted by the EEG electrodes by multiplying the ratio of *r*_*eeg*_/*r*_*meg*_.

(c)According to the fact that magnetic field strength follows an inverse square law, the strength of a magnetic field measured at increasing distance from the field source (*dM*_*fs*_), decay according to$1/{\left(dM_{fs}\right)}^{2}$. The signal strength of the scaled MEG sensors from step (b) was compensated by multiplying the ratio of ${\left(r_{meg}/r_{eeg}\right)}^{2}$.

(d)With the *griddata* function provided by MathWorks^@R^, the signal strength of the scaled MEG sensors from step (c) were interpolated at the query points specified by EEG electrodes.

### EEG based sFCT construction

Although there are many widely utilized EEG reference schemes, in this study, the EEG reference schemes referred in other literatures (Yao, 2001; Zhai and Yao, 2004; Nunez, 2010; Qin et al., 2010) were exploited to further compare the results from different studies. The four EEG reference schemes (REST, AR, LM, and LR) used to construct sFCTs are described below.

#### Reference electrode standardization technique

Based on the following important ground truths, Yao et al. proposed REST (Yao, 2001; Zhai and Yao, 2004; Yao et al., 2005, 2007). The reference is at an infinity point far away from brain sources such that an approximate neutral reference can be acquired. Given the transfer matrix from brain sources to scalp EEG sensors, the relationship between scalp EEG recording, and source activities can be written as,

(3) $$\begin{array}{l} M_{REST}=G_{REST}Src \end{array}$$

where, ***Src*** is a matrix referring to the active brain areas, ***G***_***REST***_ refers to the transfer matrix that enables the transformation from active sources to sensor representations, and ***M***_***REST***_ denotes the scalp EEG recordings that result from inside active sources *Src* with a reference at an infinity point. Similarly, for other reference schemes, the relationship between active sources, and scalp EEG recordings can be written as,

(4) $$\begin{array}{l} M_{REF}=G_{REF}Src \end{array}$$

where, ***G***_***REF***_ represents the corresponding transfer matrix of any original EEG reference schemes. Putting Equation (4) into Equation (3), ***M***_***REST***_ can be derived from any original EEG reference recordings.

(5) $$\begin{array}{l} M_{REST}=G_{REST}Src=G_{REST}\left(G_{{\;}_{REF}}^{+}M_{REF}\right)=T_{REST}M_{REF} \end{array}$$

where, $G_{{\;}_{REF}}^{+}$refers to the Moore-Penrose generalized inverse, and linear transformation ***T***_***REST***_ is written as

(6) $$\begin{array}{l} T_{REST}=G_{REST}G_{{\;}_{REF}}^{+} \end{array}$$

Equation (6) indicates that REST enables the transformation matrix ***T***_***REST***_ to be carried out without the necessity to acquire the real active brain sources ***Src*** (Yao, 2001). Therefore, REST can implicitly solve the EEG inverse problem with transfer matrices ***G***_***REST***_ and ***G***_***REF***_. Practically, matrices ***G***_***REST***_ and ***G***_***REF***_ can be deduced from an equivalent source distribution (ESD), which encloses all the possible neural sources. Thus, the transformation matrix in REST only relies on the characteristic of the assumed ESD, including the head model, electrode montage, original reference, and spatial geometry, rather than the actual brain-source data. In this study, the ESD was assumed to be a discrete layer of current dipoles forming a closed surface, as has been done in previous studies (Yao, 2001; Womelsdorf and Fries, 2006; Nunez, 2010; Huang et al., 2017).

#### AR reference, LM reference, and LR reference

Theoretically, to obtain an arbitrarily “zero level” electric potential, the EEG reference electrodes are well advised to be placed on a presumed “inactive” zone. According to variable clinical applications, in addition to REST, three other EEG reference schemes are widely used, differing on where the reference electrodes are placed. These include AR reference, LM reference, and LR reference. AR reference uses the average signal from all electrodes (Lehmann et al., 1998; Hesse et al., 2004; Huang et al., 2017). LR uses the signal from the right earlobe, and LM utilizes the mean signal from the left and right earlobes as the reference (Yao et al., 2005; Huang et al., 2017).

### Metrics of measuring similarity between sFCTS

#### Relative error

RE can represent the sum of the difference between two matrices, thus is generally utilized to evaluate the effectiveness of each reference scheme. It is calculated as:

(7) $$\begin{array}{l} RE=\frac{\left||Coh-Coh_{*}|\right|}{\left||Coh|\right|} \end{array}$$

where, *Coh* represents the coefficient matrix of IC with an infinite far-away point as the referenced node. Coh_*_ denotes the imaginary part of coherency-coefficient matrix with real measured reference schemes, including AR reference, REST reference, LM reference, and LR reference. The matrix norm ||·||is the Frobenius norm defined as following.

(8) $$\begin{array}{l} ||Coh||=\sqrt{\overset{N}{\underset{i=\text{1}}{\sum }}\overset{N}{\underset{j=\text{1}}{\sum }}Coh_{ij}^{\text{2}}} \end{array}$$

where, *N* denotes to the total electrode number, and $Coh_{ij}^{\;}$ refers to the coherency between EEG electrodes *i* and *j*. The range of *RE* should range from 0 to 1, with a smaller *RE* corresponding to greater similarity between two IC matrices.

The square and sum operations in Equation (8) enable RE measure the global difference between two IC matrices. However, the relationship between two corresponding nodes sometimes cannot be accurately determined. Therefore, additional effective metric should be considered for evaluating sFCT. Here, the overlapping rate (OR) and hamming distance (HD) are also induced to jointly assess the similarity of topographies.

#### Overlapping rate between topographies

Overlapping Rate (*OR*) detects the shared connective edges from all possible edges. *OR* was defined as the ratio of

(9) $$\begin{array}{l} OR=\frac{G_{1}⋂G_{2}}{G_{1}⋃G_{2}} \end{array}$$

where, *G*_1_ and *G*_2_ refer to two topographies, respectively. ⋂ denotes the intersection of *G*_1_ and *G*_2_, which refers to the communal edges of two topographies. ⋃ refers to the union of *G*_1_ and *G*_2_, which represent all the possible edges appeared in two topographies. From the perspective of topography, *OR* is able to detect where the edges of two topographies overlap, thus evaluating how similar the two topographies are. Higher *OR* value indicate greater similarity between two FCTs.

#### Hamming distance between topographies

Besides similar partitions, differences in partitions also contribute significantly toward evaluating how similar two topographies are. Hamming Distance (HD) is an excellent complement for *OR* because it can detect differences between two topographies. HD calculates the distance between topographies by measuring the vector entries that differ (Makram Talih, 2005; Medkour et al., 2010; van Wijk et al., 2010). The number of elements of two topographies *G*_*1*_ and *G*_*2*_ with adjacency matrices *N*^(*1*)^and *N*^(*2*)^ that disagree is calculated as follows,

(10) $$\begin{array}{l} HD\left(G_{\text{1}},G_{\text{2}}\right)=\overset{N}{\underset{i\neq j}{\sum }}\left[N_{ij}^{\left(\text{1}\right)}\neq N_{ij}^{\left(\text{2}\right)}\right] \end{array}$$

where, the square brackets notation here reflects an indicator function that is equal to one if its argument is true and zero otherwise. Smaller HD values correspond to greater similarity between two topographies.

### Simulation tests

#### Simulated dipolar sources setting

In this study, a concentric three-layer sphere model (Rush and Driscoll, 1969; Yao, 2001; Qin et al., 2010) were utilized to analyze the simulated EEG data. The radii of the three concentric spheres are 0.87(inner radius of the skull), 0.92 (outer radius of the skull) and 1.0 (radius of the head), and the conductivities are 1.0 (brain and scalp) and 0.0125 (skull) (Rush and Driscoll, 1969; Yao, 2001; Qin et al., 2010). A single equivalent sphere (Sarvas, 1987; Huang and Mosher, 1997) was exploited to model simulated MEG recordings in this study. The radius of the single sphere is 0.87(inner radius of the skull). The forward solution of MEG was generated according to Sarvas (1987).

To conveniently compare sFCTs from MEG and EEG, the simulated MEG and EEG models share the same Cartesian coordinate system, the same equivalent source distribution model, and the same electrode montage. Both EEG and MEG head models take the center of the sphere(s) as the coordinate origin. Direction from the origin toward the left ear was determined as the +y axis, and the posterior–anterior direction that from the origin to the nasion was the +x axis. The +z axis was defined as the axis that was perpendicular to +x and +y axes and pointed from the origin toward the vertex. A closed spherical cap surface with radius *r* = 0.869 and a transverse plane at *z* = −0.076, that contained 1,500 nodes on the spherical cap surface, was assumed for the discrete equivalent dipole layer sources (Yao, 2001). A 129-channel system was employed in this study and the montage was the same with the EGI (Electrical Geodesics, Inc.) collecting system with 129 electrodes.

The representation of dipolar neural source orientations was defined by the spherical coordinates system (*r*, θ, ϕ), which was converted by the corresponding Cartesian coordinate system mentioned above. Where, θ is inclination, move *r* units from the origin in the zenith direction, rotate by θ about the origin toward the azimuth reference direction, and rotate by ϕ about the zenith in the proper direction. For each testing dipolar pair, the source momentums were denoted as [−0.5, 0.5, 0.1] and [0.5, −0.5, 0.1], respectively.

**Figure 2:**
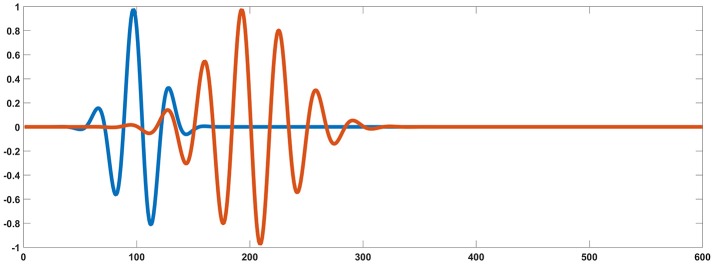
Simulated signal generated from two coherent sources (blue and orange). Based on the source settings, simultaneously collected simulated surface MEG and EEG recordings could be derived by solving an electromagnetic forward problem.

For each testing dipolar pair, the temporal process of two coherent dipolar neural sources was simulated by dot product of a Gaussian function and a cosine function, because this function has the appearance of an evoked potential (Yao, 2001) (see in Figure 2),

(11) $$\begin{array}{l} y\left(t_{i}\right)=e^{\left(-{\left(2\pi \frac{t_{i}-t_{0}}{\gamma }\right)}^{2}\right)}\;cos\left(2\pi f\left(t_{i}-t_{0}\right)+\alpha \right)\;i=1,2\cdots \;,k \end{array}$$

where, *t*_0_ = 100^*^dt, *f* = 30*Hz*, γ = 5, $\alpha =\frac{\pi }{4}$ for one dipole in the pair, and *t*_0_ = 200^*^dt, *f* = 30*Hz*, γ = 10, $\alpha =\frac{\pi }{2}$ for the other. For the two dipoles, *t*_*i*_ = i^*^dt, *i* = 1, 2, …, *k*(*k* = 600) and *dt* = 0.1.

#### One-fixed dipolar pair setting

To confirm the connections of MEG and each EEG reference scheme, the statistical results were exported from different network sparsities. The network sparsity refers to how many edges were selected from IC matrix to construct binary network. The number of the edges in binary network was determined by how many of the largest weights in the IC matrix were selected. Thus, network sparsity can be determined by how many of the largest weights were selected. In this study, the number of the largest weights ranged from 20 to 80, and the step width was set to 10. The parameters were set as follows: (1) two random selected sources were evoked with the wave format in Figure 1; (2) source orientations were set to [−0.5, 0.5, 0] and [0.5, −0.5, 0], respectively; (3) signal-to-noise ratio (SNR) was defined as the ratio of the Frobenius norm of the data matrix to that of the noise, and set to 1. Note that in the simulation EEG electrodes and MEG sensors are assumed to share the same localization to exclude the influence of electrode distribution. However, in real measurements, electrode localizations between EEG and MEG differ from each other.

#### Dipolar pairs setting

MEG is sensitive to tangentially oriented dipoles, but it is blind to the radially oriented dipoles. In contrast, EEG can sense both radial and tangential dipoles, but has trouble detecting deep source (Cohen and Cuffin, 1987). Considering that most neural activity within brain occurs in the cortex, all the dipolar pairs involved in the testing were located in upper superficial position (radius > *d* > 0.65, *d*: distance from the dipole to the center of head model) of the simulated head model (radius = 0.869). Further, each dipolar pair adopted a combined orientation with radial and tangential components. Specifically, the parameters of each dipolar pair were set as follows: (1) network sparsity ranges from 20 to 80, and the interval is set to be 10; (2) in each dipole, the two random selected sources are evoked with the wave format in Figure 1, and the corresponding source orientations were set to be [−0.5, 0.5, 0.1] and [0.5, −0.5, 0.1] respectively.

Based on the results from the one-fixed dipolar pair, the experiment with 20 dipolar pairs was designed to further validate whether the connection between MEG and EEG was repeatable, and whether the performance of each EEG reference was stable when increasing dipolar pairs. In the experiment with 20 dipolar pairs, the SNR was set to 1. Additionally, based on the results from the one-fixed dipolar pair and 20 dipolar pairs, the experiment with 100 dipolar pairs was designed to test whether the connection between MEG and EEG and the performance of each EEG reference was robust against noise. In the experiment, three SNRs (1, 2, and 5) were used.

### Real data

For real data, we used simultaneously collected MEG and EEG that were obtained during a face recognition task. These data are publicly available from the OpenfMRI website (https://openfmri.org/dataset/ds000117/). The details regarding data collection can be viewed on the hosting website (Wakeman and Henson, 2015). The pre-processing procedures are referenced by the MEG visual tutorial: Single subject in Brainstorm (http://neuroimage.usc.edu/brainstorm/Tutorials/VisualSingle). For the chosen healthy subject, the face stimuli comprised 49 famous people (23 men and 26 women). The famous faces were able to be recognized by the majority of British adults. The non-face stimuli were comprised of 50 samples from scrambled images. The details of the involved images in the face and non-face stimuli experiments can refer to the previous study (Wakeman and Henson, 2015).

Experiments have shown that a negative deflection peaking around 170 ms (N170 component) is larger when viewing faces than when viewing scrambled faces (Wakeman and Henson, 2015). A similar M170 component can be detected in MEG data (magnetometers and gradiometers) (Gao et al., 2013). Studies (Gao et al., 2013) have also shown that M170 manifests based on the global characteristics of a face, whereas the induced Gamma oscillations (30–70 Hz) are associated with the integration of visual input into a pre-existent coherent perceptual representation. Therefore, to better observe the sFCT relationship between EEG and MEG, we chose a 50–600-ms observation window and a 30–70-Hz frequency band. All of IC matrices were calculated at the given 44 Hz—which is within the gamma-oscillation range (Gao et al., 2013)—and the sparse degree was set to 50.

For cortex functional connectivity networks analysis, we relied on the minimum norm-imaging method with a dipole-orientation model and 68 scouts defined by the Desikan–Killiany atlas to construct the connection between brain areas. For scalp functional connectivity networks analysis, we employed 102 magnetometers (MAG) channels and 70 EEG channels. The 102 MAG channels were first projected to the 70 EEG channels using cubic interpolation and then sFCT analysis was conducted for inter-modality (EEG and MEG) and intra-modality (EEG based on different references) analysis.

## Results

We explored two issues using simulations. The first was an inter-modality comparison of sFCT between EEG and MEG. We expected EEG-sFCTs and MEG-sFCTs to be consistent when using the same activate sources. The second simulation compared sFCT that were based on different EEG references. We expected that a good EEG reference would result in sFCT similarity between MEG and EEG.

### Simulated data analysis

#### Fixed dipolar pair simulation

**Figure 3:**
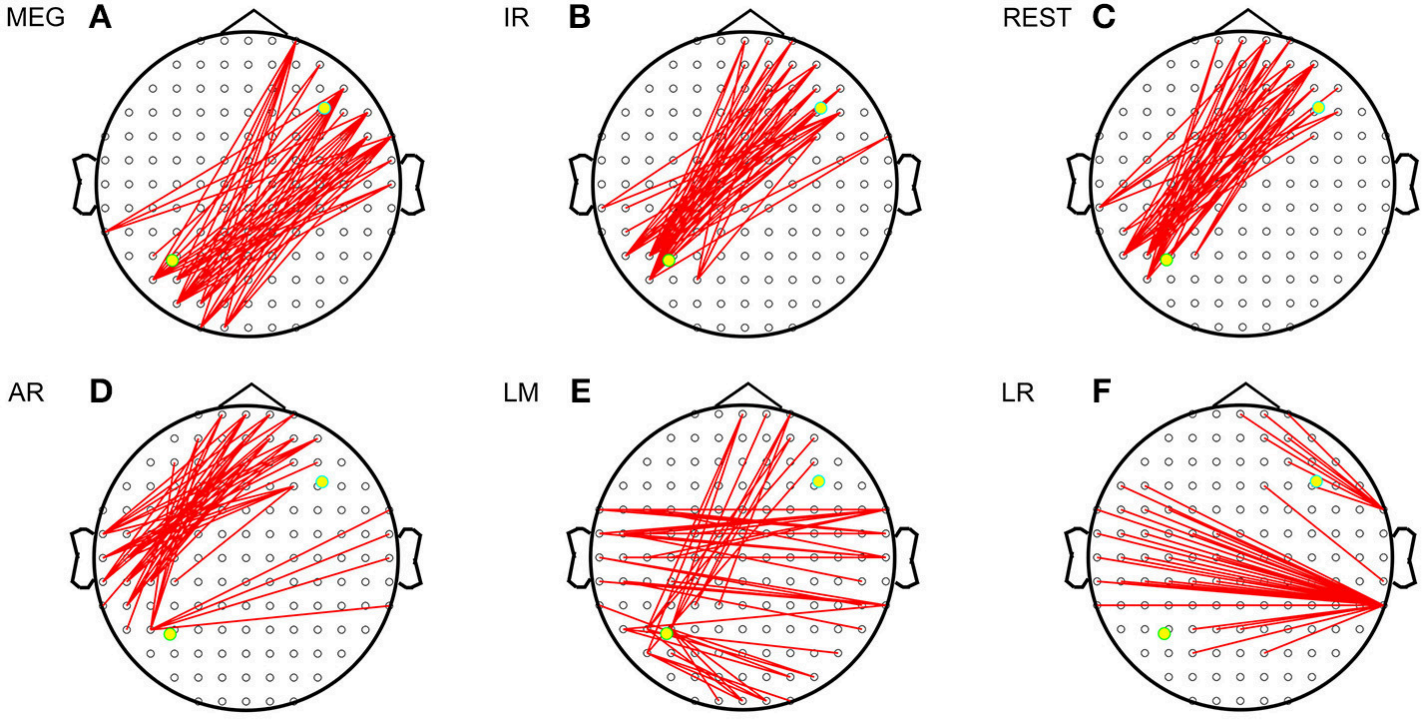
Imaginary coherence-based sFCTs derived using different methods. Network sparsity was set to 50. The red segments represent the connections between two electrodes and the two yellow dots represent the simulated sources. **(A)** sFCT of MEG. **(B)** sFCT of IR. **(C)** sFCT of REST. **(D)** sFCT of AR. **(E)** sFCT of LM. **(F)** sFCT of LR.

Figure 3 shows the sFCTs generated by each different approach by setting one fixed dipolar pair. We observed two important results. First, visual inspection shows that sFCT varied considerably among the EEG reference schemes and that similarity to MEG-sFCT was evident for some of the EEG-sFCTs. Second, among the different EEG references, REST appears have resulted in sFCTs that were the most similar to those based on IR. Our observations are in accordance with the electromagnetic theory that MEG and EEG are closely related to each other. The three metrics RE (relative error), OR (overlapping rate), and HD (hamming distance) were used to quantitatively inspect how similar two sFCTs were to the global difference, similar partition, and difference partition, respectively. Through these metrics, two types of differences were evaluated. One was the sFCT difference between each EEG reference and IR, and the other was the sFCT difference between the two modalities.

**Figure 4:**
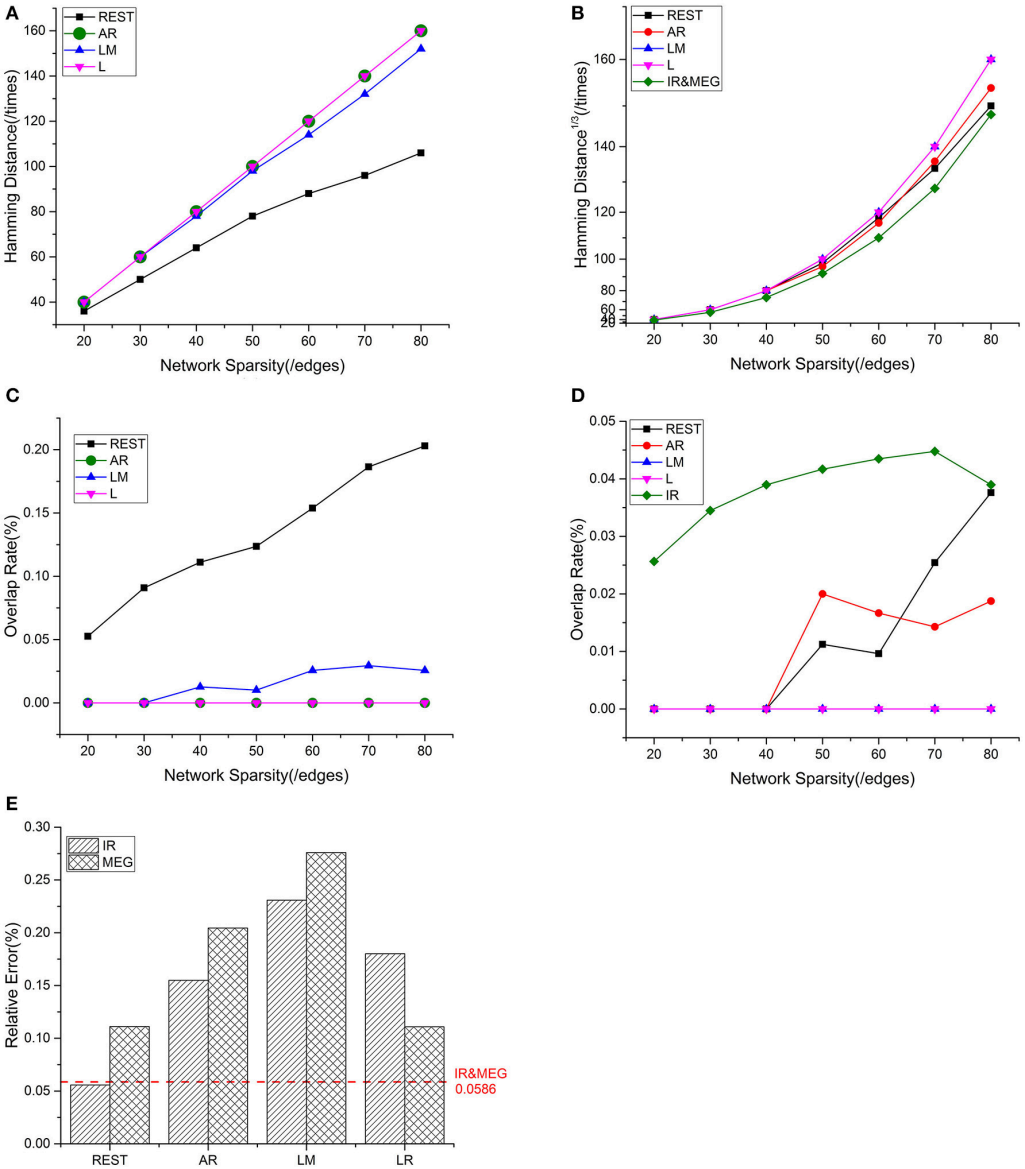
Results of one fixed dipolar pair with different network sparsities. Network sparsity ranges from 20 to 80. **(A)** HD (Hamming Distance) between each EEG reference and the EEG IR at different network sparsities. **(B)** HD between each EEG reference and MEG at different network sparsities, including the HD between EEG IR and MEG. **(C)** OR (Overlapping Rate) between each EEG reference and the EEG IR at different network sparsities. **(D)** OR between each EEG reference and MEG at different network sparsities, including the HD between EEG IR and MEG. **(E)** RE (Relative Error) between each EEG reference and two standards (MEG and the EEG IR). The red dash line is used to represent the RE between IR and MEG, which stays the same despite the change in sparsity.

Mean values for the different metrics are exhibited in Figure 4 for the intra—and inter-modality comparisons. IR-based sFCT is regarded as the standard. Compared with other schemes, REST was the best EEG reference scheme because of its much lower HD (Figure 4A). Additionally, each EEG reference and IR had the same tendency as MEG (Figure 4B). We used OR values to evaluate the same sFCT partition. As we increased network sparsity, REST always had the highest OR, meaning that for sFCT, it is the closet to IR (Figure 4C). The OR results also showed a certain connection between IR and MEG despite the network sparsity (Figure 4D). EEG-sFCT based on LM or LR rarely shared common partitions with MEG. OR for AR-based EEG-sFCT even decreased at high network sparsity. Unlike the other schemes, although the OR for REST-based EEG-sFCT was rather low at lower sparsity, at high sparsity it intersected with the OR for IR. As shown in Figure 4E, we also used RE to measure the overall difference between IC matrices based on the different approaches. The difference between each EEG reference scheme and IR, and the difference between EEG and MEG are exhibited. The red dashed line illustrates the RE difference between IR and MEG. REST performed the best among the EEG references, as evidenced by its low RE when compared with IR and MEG.

Based on these results, we can conclude that EEG and MEG are related when they are deduced from the same source. sFCTs derived from the EEG references all had different degrees of similarity with IR and MEG. Among the EEG reference schemes, REST appears to be the most desirable because it produces sFCTs that are the most similar to MEG-sFCTs and IR.

#### Statistical comparisons of sFCT on 20 dipolar pairs

**Figure 5:**
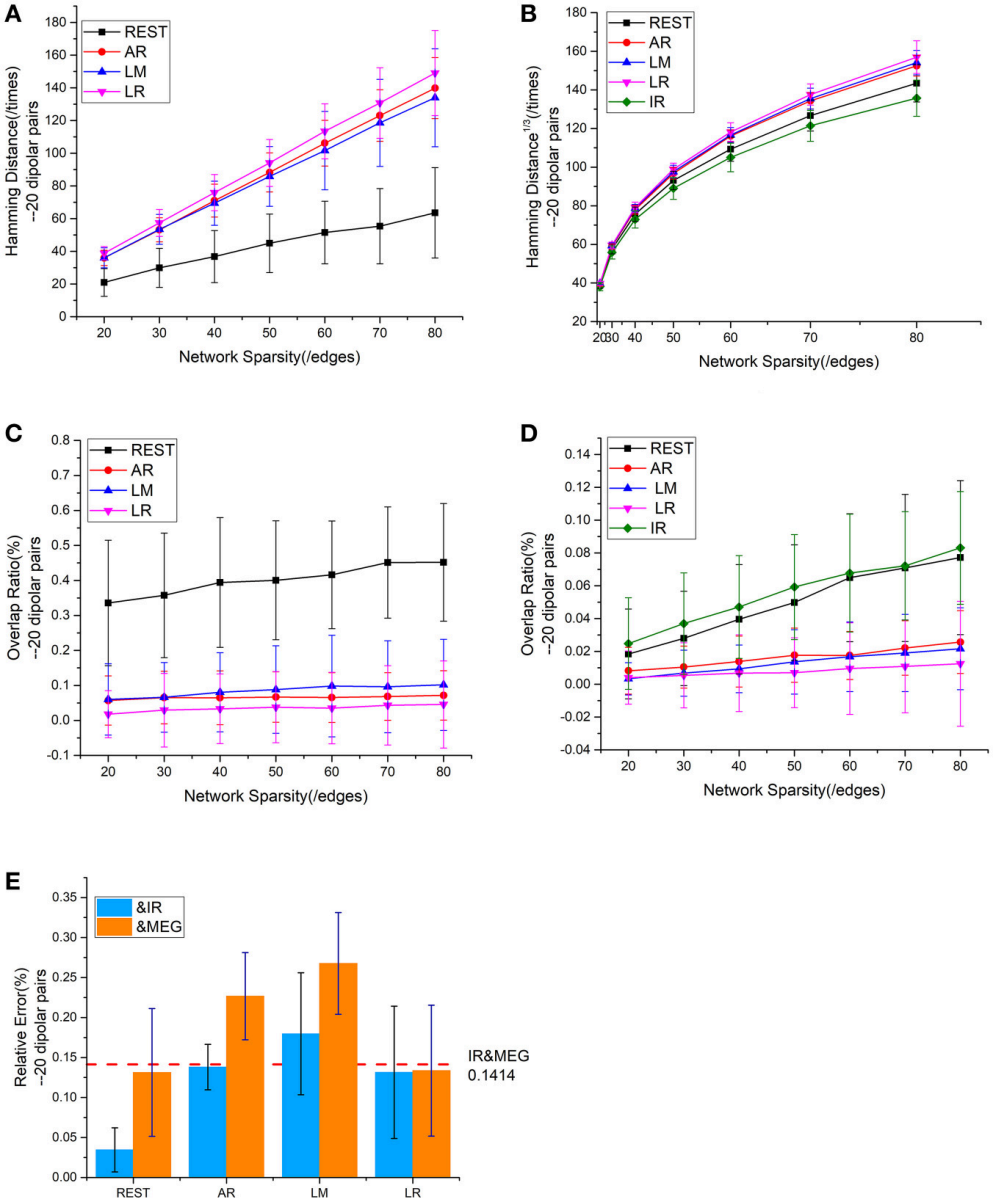
Results using 20 dipolar pairs with different network sparsities. **(A)** HD (Hamming Distance) between each EEG reference and the EEG IR at different network sparsities. **(B)** HD between each EEG reference and MEG at different network sparsities, including the HD between EEG IR and MEG. **(C)** OR (Overlapping Rate) between each EEG reference and the EEG IR at different network sparsities. **(D)** OR between each EEG reference and MEG at different network sparsities, including the HD between EEG IR and MEG. **(E)** RE (Relative Error) between each EEG reference and two standards (MEG and the EEG IR). RE between IR and MEG (the red dash line) stays the same at different network sparsities.

We employed a repeatability test using 20 dipolar pairs to further validate the relationship between EEG—and MEG-derived sFCT (Figure 5). The HD results using 20 dipolar pairs again indicate that the REST scheme has a lower transformation cost than others. The mean HD for REST only consumed 49.09% of the cost for AR, 50.62% for LM, and 45.98% for LR (Figure 5A). Moreover, despite different degrees of sparsity, EEG reference schemes share similar tendencies with MEG-based sFCT (Figure 5B). Compared with IR-based sFCT, REST was again revealed to be superior because of its significant higher OR (Figure 5C). The mean OR for REST reached 0.401, while results from the other three reference schemes are all around zero (< 0.1), which means they produced sFCTs that hardly intersected IR-based sFCTs. Additionally, OR values for REST were very similar to IR and had a certain degree of similarity with MEG-based sFCT (Figure 5D).

The analysis of the different metrics, as functions of network sparsity, presented in Figure 5 shows that differences in similarity for inter—and intra-modality comparisons. Compared with other EEG references, sFCTs derived from REST-based EEG are most similar to IR and MEG-based sFCTs. To evaluate the reference effects statistically, we performed a univariate analysis of variance (UANOVA) on the HDs, ORs, and REs from inter—and intra-modality experiments. UANOVA results were corrected for multiple comparisons using Bonferroni's *post*-*hoc* test and *p* < 0.05 was considered statistically significant.

We found significant differences for sFCT similarity (each of the three metrics) among the involved EEG reference schemes. (HD: *F* = 1156.73, *p* < 0.001; RE: *F* = 1076.49, *p* < 0.001; OR: *F* = 1616.98, *p* < 0.001). Additionally, pair-wise multiple comparisons reveal significant differences (Bonferroni's test, *p* < 0.05) for all comparisons except for AR vs. LR for the RE metric (HD: *p* = 0.038 for AR vs. LM, p < 0.001 for all other pairs; RE: *p* = 0.073 for AR vs. LR, *p* < 0.001 for all other pairs; OR: *p* = 0.011 for AR vs. LM, *p* < 0.001 for all other pairs).

We also found significant differences for each metric when comparing EEG-sFCT (including different references and IR) with MEG-sFCT (HD: *F* = 465.43, *p* < 0.001; RE: *F* = 728.96, *p* < 0.001; OR: *F* = 490.56, *p* < 0.001). Although pair-wise multiple comparisons revealed many significant differences (Bonferroni's test, *p* < 0.05), those for AR vs. LM for the OR metric and REST and LR vs. IR and for the RE metric did not significantly differ (HD: *p* = 0.029 for AR vs. LM, *p* < 0.001 for all other pairs; RE: *p* = 1.00 for REST vs. IR, *p* = 0.394 for LR vs. IR, and *p* < 0.001 for all other pairs; OR: *p* = 0.122 for AR vs. LM, *p* = 0.006 for LM vs. LR, and *p* < 0.001 for all other pairs).

#### Results from 100 dipolar pairs with different noise levels

**Figure 6:**
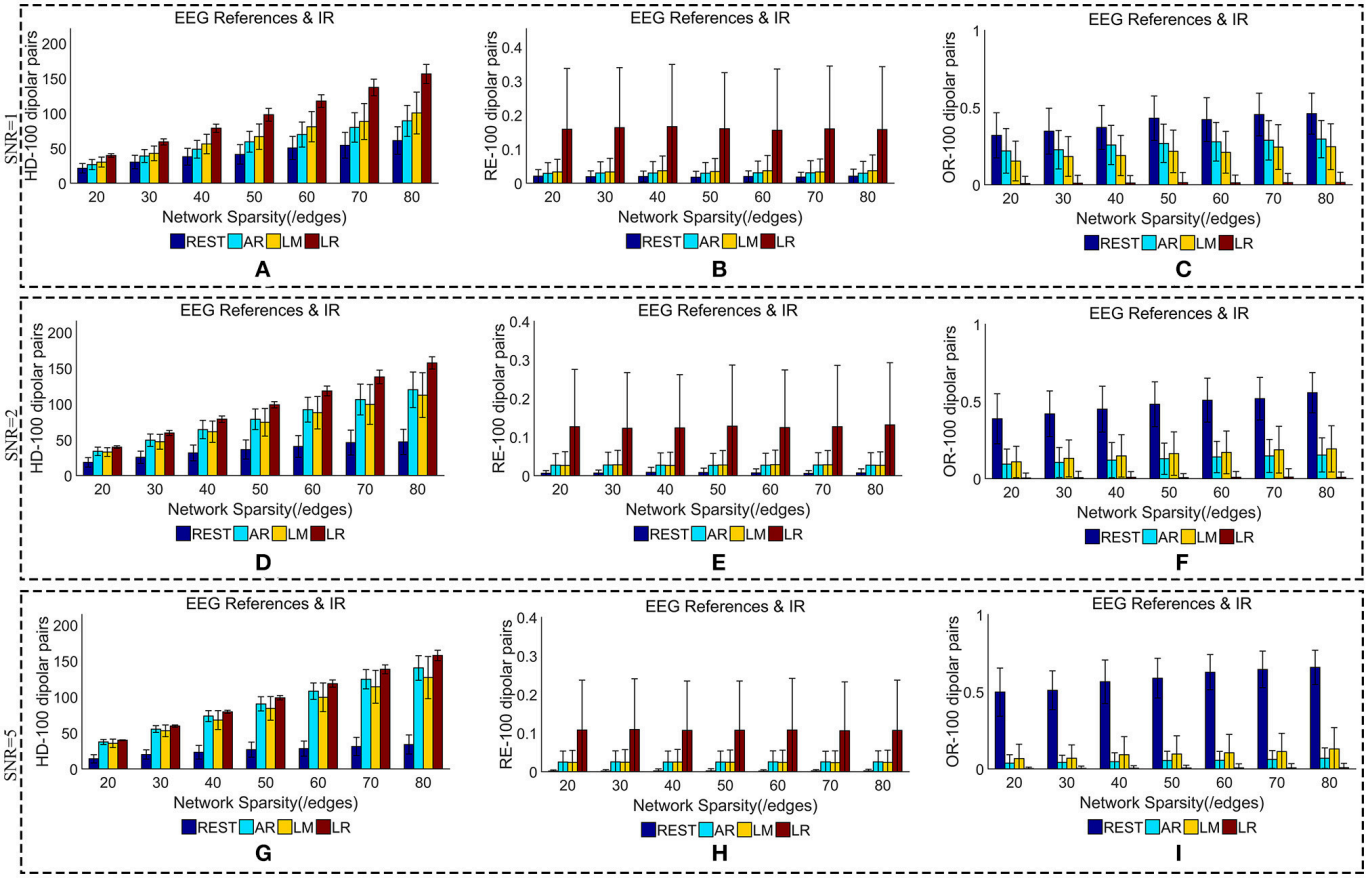
Similarity of sFCTs between EEG references (REST, AR, LM, and LR) and IR based on HD (Hamming Distance), RE (Relative Error), and OR (Overlapping Rate) with various SNR (from 1, 2 to 5). In the case of SNR = 1, different metrics, HD **(A)**, RE **(B)**, and OR **(C)**, were used to measure similarity. In the case of SNR = 2, HD, RE, and OR correspond to **(D–F)**, respectively. Similarly, in the case of SNR = 3, HD, RE, and OR correspond to **(G–I)**, respectively. Each bar graph gives results from the four EEG references (REST, AR, LM, and LR) under different network sparsities (ranging from 20 to 80 with an interval of 10).

**Figure 7:**
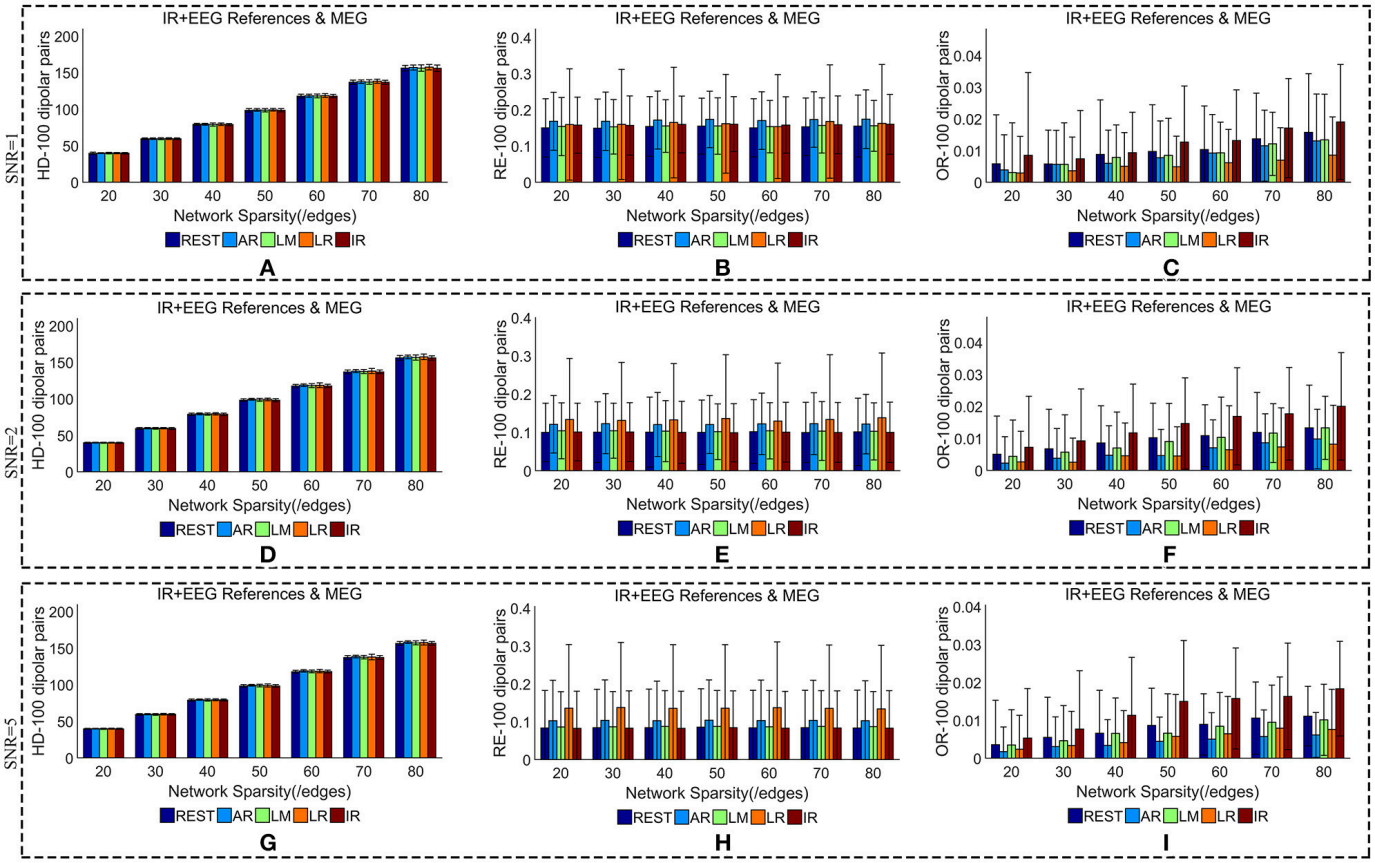
Similarity of sFCTs between EEG (REST, AR, LM, LR, and IR) and MEG based on HD (Hamming Distance), RE (Relative Error), and OR (Overlapping Rate) with different SNRs (1, 2, and 5, respectively). In the case of SNR = 1, different metrics, HD **(A)**, RE **(B)**, and OR **(C)**, were used to measure similarity. In the case of SNR = 2, HD, RE, and OR correspond to **(D–F)**, respectively. Similarly, in the case of SNR = 3, HD, RE, and OR correspond to **(G–I)**, respectively. Each bar graph gives results from the four EEG references (REST, AR, LM, LR) and IR under different network sparsities (ranging from 20 to 80 with an interval of 10).

We added different noise levels to the simulated EEG/MEG recordings to test the robustness of each EEG reference against noise. Figures 6, 7 illustrate the quantitative metrics obtained from 100 dipolar pairs under different SNRs (SNR = 1, 2, or 5). Figure 6 shows the results from the comparison between EEG references and Figure 7 presents the EEG/MEG comparisons.

The HD between each EEG reference and IR slightly decreased as SNR increased for a given network sparsity (Figures 6A,D,G). At each sparsity and SNR combination, REST had the lowest HD compared with the other EEG references. Similarly, the RE between each EEG reference and IR decreased as SNR increased (Figures 6B,E,H). In particular, as SNR increased, RE for REST became extremely small. When SNR was set to five, the average REs for REST were almost zero (0.002) for each given network sparsity. Compared with the other reference schemes, REST also had the lowest RE for each given network sparsity and SNR combination, meaning that REST is also the best reference in the terms RE. The OR between REST and IR increased as SNRs increased (Figures 6C,F,I), while OR resulting from AR, LR, or LM decreases as SNR increased for each given network sparsity. Higher OR indicates more similarity between two sFCTs, which means that REST was better at recovering IR from the perspective of sFCT. Compared with the other three EEG reference schemes, the OR for REST was the highest for each given network sparsity and SNR combination. Therefore, in the terms of HD, RE, and OR, REST is the most robust metric against noise, while the other three EEG reference methods appear easily affected by noise.

When we compared MEG-sFCT with each EEG-sFCT (including IR-sFCT), we found that HDs were stable as SNR increased for each given network sparsity (Figures 7A,D,G). Overall, no obvious differences were observed in the cost of transforming each EEG-based sFCT to an MEG-based sFCT for any of the network sparsity and SNR conditions (Figures 7A,D,G). The RE between EEG and MEG decreased slightly as SNR increased (Figures 7B,E,H). In particular, for each given network sparsity and SNR condition, the REs for REST—and IR-based sFCTs were lower than those for sFCTs based on other EEG references, meaning that REST and IR are comparable in the terms of RE, while the other three EEG reference schemes are not. The OR between EEG and MEG did not increase with SNR. At different noise levels, IR shared the highest OR with MEG-sFCT for each given network sparsity. Compared with AR, LM, and LR, the results from REST-based sFCTs were closer to those based on IR for each given network sparsity and SNR condition. Therefore, in the terms of HD, RE, and OR, the similarity between MEG-based sFCT and EEG-based sFCT is robust against noise. Additionally, aside from IR, REST is the EEG reference scheme that is most similar to MEG for each given network sparsity and SNR.

### Real data analysis

The simulation results indicated that EEG—and MEG-based sFCTs are related. This is likely because both EEG and MEG are sensitive to the same generators (synchronous post-synaptic currents in aligned pyramidal cells). To validate the simulation predictions, we performed the same analyses on real measurements.

**Figure 8:**
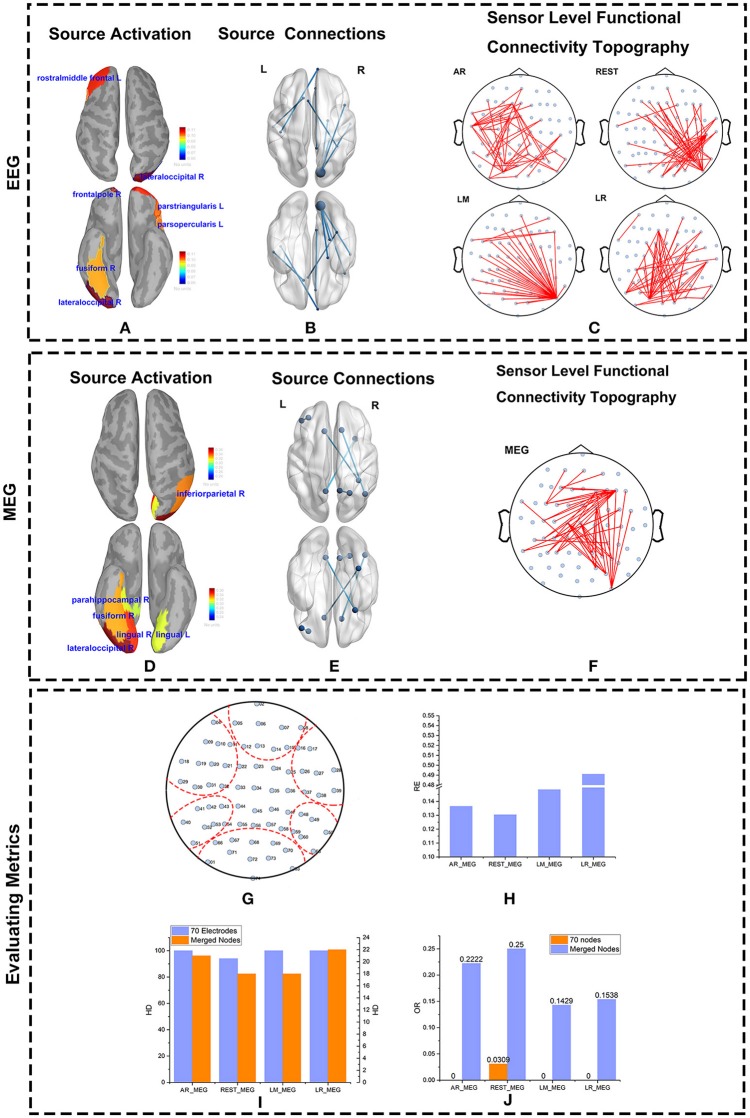
Source and Scalp results from real data. The source activity for MEG and EEG were both reconstructed using Minimum Norm Imaging and model Dipole Orientations. The brain was divided into 68 scouts according to the Desikan-Killiany atlas. The source activity, source connections, and scalp functional connectivity topographies evaluated by EEG and MEG are illustrated. **(A,D)** Reconstructed source activity. The cortical activity is shown from above and below. The active areas are colored and labeled. **(B,E)** Connections at the source level from EEG **(B)** and MEG **(E)** are represented. The source connections are shown from above and below. The blue nodes represent scouts embedding strong activity. The blues lines denote the connections between two scouts. **(C,F)** Sensor-level functional connectivity topographies (sFCTs) from the four different reference schemes **(C)** and MEG **(F)** are represented. The blue nodes denote the distribution of electrodes, and the red lines represent the connections between electrodes. All sFCTs were constructed with network sparsity set to 50 edges. **(G–J)** Metric evaluations of seven electrode groups. **(G)** Electrode groups are shown encapsulated by the red dotted lines. **(H)** The RE (Relative Error) between each reference and MEG. **(I)** The HD (Hamming Distance) between each reference and MEG. **(J)** The OR (Overlapping Rate)between each reference and MEG.

The major active areas in the brain as determined by the EEG and MEG data were similar (Figures 8A,D). Similarly, results from the IC matrix comprising 68 scouts (defined by the Desikan-Killiany atlas) indicate that EEG and MEG recordings identify mostly the same brain connections when viewing faces (Figures 8B,E). Both MEG and EEG correctly identified the core active regions distributed in the ventral occipito-temporal cortex, known as the fusiform, and occipital face areas (Haxby et al., 2000; Ishai et al., 2005; Kanwisher and Yovel, 2006; Barbeau et al., 2008; Blank et al., 2014). Other regions, such as those in the frontal cortex, were also active during face recognition (Blank et al., 2014). Although sFCT usually appears a little different from the cortical connections because of the volume conduction effect, the active regions at the cortex level should be consistent with those at the source level. MEG-based sFCT reflected the prominent active regions at the source level (Figures 8C,F). Even though the differences in sFCTs generated by the varying EEG reference schemes is visible, all of them reflect the major active cortical regions to a certain degree. Considering the projection errors, REST-based sFCT can still identify the major connections of important nodes. Although LM-based sFCT could identify the activity in the occipital lobe, it missed the activation in the temporal lobe. In this trial, AR—and LR-based sFCTs were concentrated on activity in the left hemisphere, which may lead to a misleading identification of activation at the source level.

As with the simulations, we quantified sFCT similarity of the real measurements by using the RE, HD, and OR metrics. Note that for real data, all the metrics are calculated on merged nodes for two major reasons. First, when brain activity is transmitted from cortical neurons to the electrodes on the scalp, influence from factors such as volume conduction is inevitable. Second, in real measurements, the distributions of EEG and MEG on the electrodes are different. Although we mapped MEG sensors to the corresponding EEG electrodes by coordinate transformation, some level of bias cannot be avoided.

To better visualize the connection among active scouts at scalp-level, 70 electrodes were divided into seven groups (Figure 8G), and the following similarity calculations for RE, HD, and OR are based on the group-level data. Each of the seven groups corresponds to a functional specialized scout. For example, electrodes marked with 02, 05, 06, 07, 08, 12, 13, 14, 15 belong to the frontal lobe group. In terms of RE vs. MEG, the best EEG reference scheme was REST, as evidenced by its lower RE values (Figure 8H). Similarly, the HD between EEG—and MEG-sFCTs was lowest for the REST and LM reference schemes (Figure 8I). Thus, the cost of transformation was less for REST and LM than for the other two references. The OR values for REST-based sFCTs were higher than those for the other reference schemes, indicating a greater degree of overlap with the MEG-based sFCTs (Figure 8J). Therefore, in terms of HD, RE, and OR, if we take MEG-sFCT as the standard, REST-based sFCT is the most similar among the four EEG references.

## Discussion

Clinically, abnormal functional connectivity in human cortex is thought to be implicated in several diseases. Information processing in the brain can be elaborated in the form of sFCTs derived from imaginary coherence matrixes. Therefore, studying sFCT has an essential and profound impact on the field of neuroimaging. For EEGs, scalp recordings can only provide the potential difference between two points because nothing in the body has a potential of zero. Thus, the use of an appropriate reference is vital, and all reference choices inevitably affect the EEG measurements. The reference issue is therefore always a research topic that is rooted in the hardware design for EEG detection, and researchers have been searching for the best reference scheme for quite some time. Additionally, the IR of an EEG cannot be detected in real measurements. Because of this, when measuring real brain activity, the results provided by different EEG references cannot be evaluated against any gold standard. However, MEG is a reference-free methodology that can be used to collect real measurements. According to the principles of electromagnetics, EEG and MEG should be related, and the two modalities tend to produce similar sFCTs given the same brain activity. Therefore, similarity to MEG-sFCT is likely to be a good metric for accessing the performance of EEG reference schemes.

In our simulation experiment, all EEG-based sFCTs had a similar tendency with respect to MEG-sFCT at different network sparsities, which indicates that EEG-sFCT and MEG-sFCT are strongly linked given the same active source. In particular, MEG and the EEG IR produce highly similar sFCTs. Therefore, we infer that in real measurements, MEG may be a good substitute for the EEG IR. Furthermore, the suitability of the EEG reference scheme can be determined by the MEG obtained from real data. In the future, MEG-based sFCT may not only be used to evaluate the performance of different EEG reference schemes, but might be taken as a new reference scheme by transformation to EEG-sFCT. Particularly, compared with references like AR, LR, and LM, REST-based sFCT appears to have good stability when varying dipole number and exhibits robustness to different noise levels. In the intra-modality experiment, REST-based sFCT exhibited only minor differences from IR-based sFCT. In the inter-modality experiment with MEG-sFCT as the reference, REST exhibited a comparable similarity with IR-based sFCT. Thus, MEG and EEG do produce sFCTs with a certain degree of similarity, and among the tested EEG reference schemes, REST is the most consistent with both IR and MEG.

Because the EEG IR cannot be detected in real measurements, MEG-sFCT was used as the standard for evaluating different EEG reference schemes in the face-recognition data analysis. Theoretically, if conducting error is ignored, the same source activation should product the same sFCT. On the whole, even when affected by field spread and volume conduction, EEG—and MEG-based sFCTs overlap considerably. Essentially, a good sFCT is thought to recover real activation at the source-level. Compared with other reference schemes, sFCT from REST can reflect brain activity relatively well, with the same accuracy as MEG. In contrast, LM only accounts for some of the prominently active regions, and AR and LM cannot localize activity well at all. These other methods thus have a rather negative influence on evaluating brain activity with sFCT.

Compared to other reference methods, our experiments show that REST produces sFCT that is more consistent with IR—and MEG-derived sFCT. Therefore, we recommend using REST when analyzing EEG-based sFCT. Moreover, REST is an appropriate substitute for MEG for using sFCT to inspect brain activity. Here, we confirmed the relationship between IR and MEG with three metrics, but we did not quantize the sFCT transformation between IR and MEG. Further study must figure out the mathematic details of the transformation from MEG to EEG, especially the EEG IR for real measurements. This will allow research from different institutes to be unified.

One highlight of our study is, for the first time, to study sFCT between EEG and MEG. Many studies developed exhaustive analysis, based on the new EEG reference scheme—REST provided by Yao (Qin et al., 2010; Zheng et al., 2018), including the analysis of the EEG reference effects on brain network (functional connectivity) (Chella et al., 2016). However, these studies only were limited to discussions on the difference among EEG reference schemes and did not take the sister modality of EEG—MEG into account. Considering the close tie between EEG and MEG and the characteristics that MEG is reference free, we not only compared the difference of sFCT among EEG reference schemes but also gave deep insight into the commonality and difference between EEG-based and MEG-based sFCTs. By building bridges between EEG and MEG in the term of sFCT, we wish our study could provide a reference for the studies in the field of EEG reference schemes, as well as on the relationship between EEG and MEG.

## Limitation

Both MEG and EEG originate from the neurobiological activity within the brain. MEG detects the surface magnetic fields while EEG detects scalp electrical potential. Generally, EEG and MEG detection should be complementary, and the methods have similarities as well as differences. To a certain degree, this study neglects the differences between EEG and MEG with respect to sFCT, and mainly focuses on the similarity between the two modalities. The results from the simulated data indicate that sFCTs based on MEG are indeed similar to those based on EEG. However, the employed simulation model is merely an approximation of a real head model, and the combination of active sources in simulations is far simpler than that in real brain activity. Therefore, results from the real-data analysis show that the degree of similarity between EEG-sFCT and MEG-sFCT based is actually lower than what we observed using simulation data.

Besides, only two dipoles the minimum dipolar pair, with randomly positions, and fixed orientation in the upper hemisphere are used to simplify our simulation on brain functional connectivity. We will, in the future work, take the influence of the orientation of dipoles and the number of involved dipoles into consideration to gain deeper insight into the connection between EEG references and MEG on sFCT. Further, except IC, weighted phase lag index also proved to be insensitive to volume conduction (Vinck et al., 2011), we will consider to introduce such kind of index to analyze the connection between EEG reference and MEG. Our study on the similarities between MEG and EEG is likely to be taken as a reference for other researchers and might inspire more related studies. We will also conduct further research on this issue.

## Conclusion

sFCT can help physicians analyze abnormal brain activity. sFCTs based on different EEG references or on MEG are similar to varying degrees. The reference issue is fundamental for scalp EEG-based studies, and the choice of reference scheme is usually based on the subjective judgment of the researchers, which may differ from one to the other. By investigating the relationships between sFCTs based on different references and modalities, we found the most appropriate EEG reference scheme. Simulated data with dipolar pairs indicated similarities between sFCTs that were derived from different EEG references, as well as similarities between EEG—and MEG-derived sFCTs. Quantized numeric evaluations showed that REST-based-sFCT is the most similar to that derived from MEG or IR. Thus, the REST-based reference had the best performance among four EEG reference methods that we tested. Furthermore, when we analyzed EEG and MEG data obtained when people viewed faces, the REST-derived sFCT was again the most similar to MEG-sFCT. Although this study has shown that sFCTs derived from the two modalities are similar, we must remember that the two are indeed different, and a completely accurate model relating EEG to MEG remains to be determined.

## Author contributions

YH: executed experiments and wrote the manuscript; JZ and QL: conceived and planned the overall experimental design, supervised experiments, and improved the manuscript; YC and GY: helped manuscript corrections; GFY: contributed to the manuscript correction.

### Conflict of interest statement

The authors declare that the research was conducted in the absence of any commercial or financial relationships that could be construed as a potential conflict of interest.
